# Extent and activity of adult hippocampal neurogenesis

**DOI:** 10.3389/fnins.2025.1709208

**Published:** 2025-11-21

**Authors:** Ryan K. Betters, Robert W. Manly, Pearl M. Huynh, Elif Tunc-Ozcan

**Affiliations:** 1 Department of Neurosciences, University of New Mexico, Albuquerque, NM, United States; 2 School of Medicine, University of New Mexico, Albuquerque, NM, United States

**Keywords:** adult hippocampal neurogenesis, dentate gyrus, subventricular zone, immature neuron activity, chronic stress, aging

## Abstract

Our understanding of adult neurogenesis has advanced far since Joseph Altman reported newborn neurons in the adult rodent brain over 60 years ago, but only recently have we been able to directly interrogate its role in the brain. While the olfactory-associated neurogenesis seen in the subventricular zone of many other mammals is greatly diminished in humans, hippocampal neurogenesis persists and may have important roles for learning, memory, and emotional regulation. A reduction in neurogenesis may play a role in neurodegenerative disease, and recent studies have suggested that hippocampal neurogenesis counteracts age-associated cognitive decline. Neurogenesis is also negatively impacted by chronic stress, a major contributor to a wide variety of psychiatric disorders. Interestingly, antidepressants have been shown to enhance neurogenesis, and some of their behavioral effects are driven by newborn neurons in the dentate gyrus of the hippocampus. Little is known, however, about how these newborn neurons integrate into and contribute to the neural circuitry of the hippocampus. Recent evidence suggests that the behavioral implications of neurogenesis are driven by the activity of newborn neurons, which may or may not be coupled to the extent of cell proliferation. This review discusses what is known about these two elements of neurogenesis, and how a complete picture of both is necessary to fully understand their physiological implications.

## 1 Introduction

In 1962, Dr. Joseph Altman published a report in Science asking a simple question that would go on to become one of the most contested topics in neuroscience for decades to come; are new neurons formed in the brains of adult mammals (Altman, 1962)? Altman’s report presented the first hard evidence of neurogenesis in the adult mammalian brain, which was considered by most neuroscientists at the time to be either nonexistent or, at best, vestigial and physiologically irrelevant. Indeed, neurogenesis was an unexpected observation in Altman’s (1962) study which focused on glial involvement at sites of traumatic brain injury, but the histology was clear; the cell proliferation marker thymidine-H3 labeled both glia and neurons. Altman recognized that any conclusions, however tentative, in favor of adult neurogenesis were likely to be rejected by the field at large without extensive evidence. To that end, he conducted thorough autoradiographic studies that confirmed neurogenesis in the mature rat and cat brain (Altman, 1963; Altman and Das, 1965a), culminating in Nature where their evidence was consolidated into a single, highly persuasive publication (Altman and Das, 1965b). Altman’s work, while widely recognized today as seminal, was largely dismissed at the time despite his efforts. Though most neuroscientists eventually came to accept the existence of adult neurogenesis in some mammals, there remained two major points of contention that drive research to this day: what is the true extent of adult human neurogenesis; and what are the functional consequences of these newborn neurons?

**FIGURE 1:**
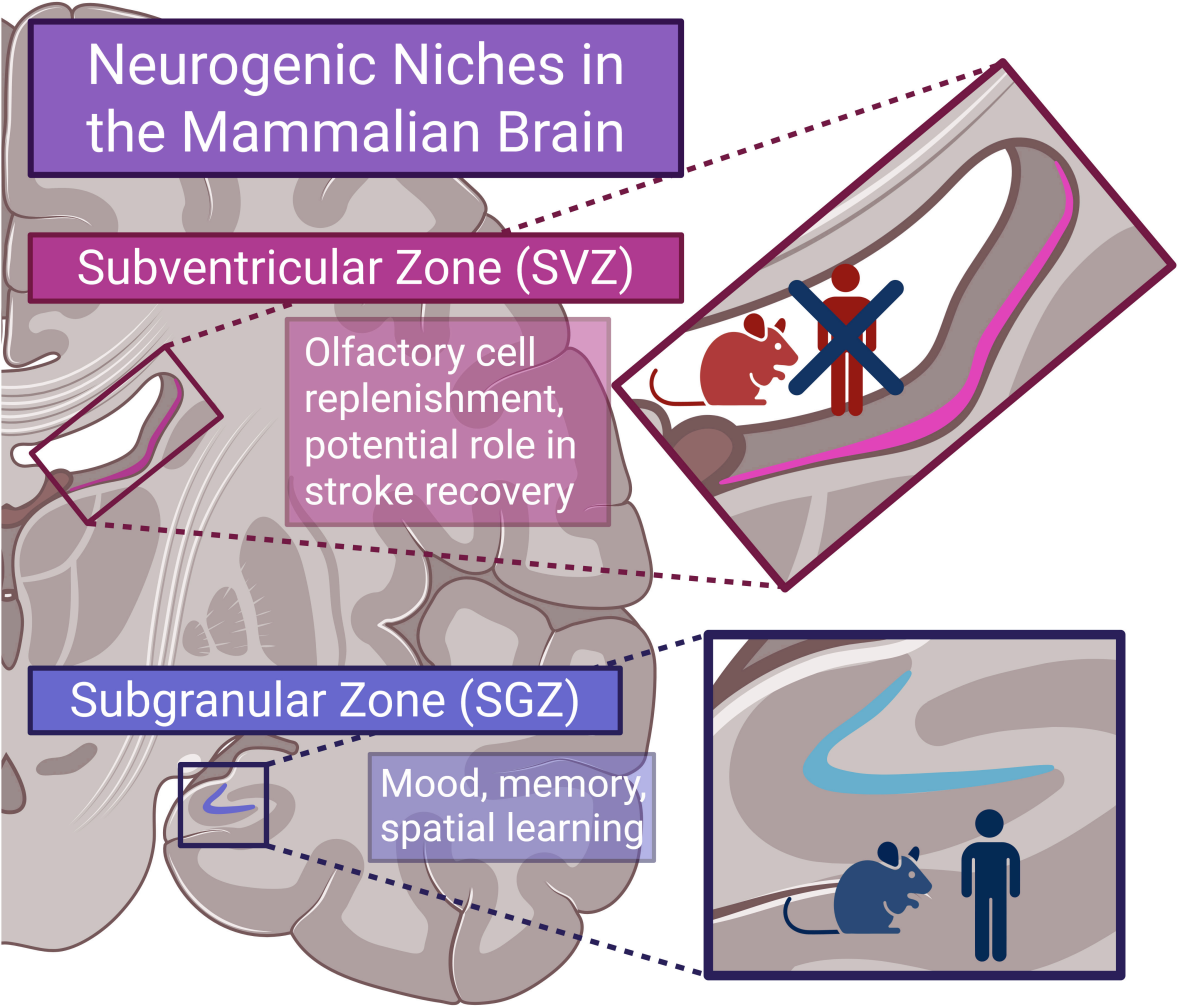
Neurogenic niches in the mammalian brain. Adult mammalian neurogenesis generally occurs in the subventricular zone (SVZ) and subgranular zone (SGZ) but is limited to the SGZ in humans where it contributes to mood, memory, and spatial learning. Created in BioRender. Betters, R. (2025) https://BioRender.com/y4lyord.

Some of the first strong evidence for adult human neurogenesis came from Eriksson et al. (1998), a cell birthdating study that used postmortem brain tissue of cancer patients that had previously received bromodeoxyuridine (BrdU) injections, a thymidine analog that incorporates into the DNA of dividing cells, to stage their tumors. BrdU birthdating had been well-established in animal studies to identify neurogenic brain regions, but Eriksson et al. (1998) extended the method to humans and reliably identified newborn BrdU-positive cells in the hippocampus. Eriksson et al. (1998) noted that these newborn cells were limited to the subgranular zone (SGZ) in the dentate gyrus of the hippocampus and expressed varying levels of neuronal markers; approximately 22% expressed NeuN, 23% expressed neuron specific enolase (NSE), and 8% expressed Calbindin. Regarding glial identity, 18% of the BrdU-positive cells in the dentate gyrus expressed glial fibrillary acidic protein (GFAP) and had typical astrocytic morphology (Eriksson et al., 1998). The study concluded that the population of BrdU-positive cells negative for both neuronal and glial markers were likely “quiescent undifferentiated cells” and/or an unexamined phenotype (Eriksson et al., 1998). Supporting evidence in the subsequent years would paint a more complete picture of the neurogenic regions in the human brain using cell proliferation markers (Knoth et al., 2010; Dennis et al., 2016; Liu et al., 2008) and primary stem-cell isolation (Palmer et al., 2001); compared to the extensive neurogenic activity seen in the SGZ and subventricular zone (SVZ) of the rodent brain, human neurogenesis is modest and generally restricted to the SGZ (Figure 1). Newborn neurons in the rodent SVZ (and many other non-human mammals) migrate along the rostral migratory stream (RMS) to replenish and potentially modulate the olfactory bulb (Lim and Alvarez-Buylla, 2016). Despite this being a lifelong process in most mammals, human SVZ neurogenesis is greatly diminished and has no clear physiological function besides a potential role in ischemic stroke recovery (Zhang et al., 2008). There is clinical potential for SVZ-associated stem cell therapy, and the SVZ is a potential target for preventing and treating glioblastoma (Kim et al., 2016; Altman et al., 2019).

There is strong evidence for human hippocampal neurogenesis occurring well into adulthood, albeit at a steadily decreasing rate (Boldrini et al., 2018), but we lack a cohesive scientific discourse surrounding its physiological role, particularly the relationship between neurogenic extent and activity. Research emphasis is generally on the former, relying on the assumption that the number of newborn neurons sufficiently explains any functional implications. This approach ignores the reality that individual neurons vary drastically in activity, even in otherwise identical cell populations. This review focuses on the relationship between the extent of neurogenesis and activity of the newborn neurons themselves, with a particular emphasis on how we might use this information to inform future studies.

## 2 What is adult hippocampal neurogenesis?

Adult hippocampal neurogenesis is the process by which new neurons are generated in the dentate gyrus of the hippocampus in the adult brain. The subgranular zone of the dentate gyrus is the only region of the hippocampus where new neurons are continuously produced throughout life in all mammalian species thus far tested (Figure 1; Baptista and Andrade, 2018). The generation of new neurons is a hierarchical, activity-dependent process that starts with radial glia-like precursors that quickly transition to progenitors before eventual differentiation into neuroblasts. This immature neuronal population matures and migrates a short distance from the subgranular zone of the dentate gyrus to the granular layer, where it integrates into pre-existing circuits (Kempermann et al., 2015). As a part of the hippocampal circuitry, adult-born neurons are more excitable than mature granule neurons and display elevated long-term potentiation (Ge et al., 2007), which has been proposed to provide them with privileged roles in some forms of hippocampal-dependent functions, such as memory consolidation, spatial and temporal pattern separation, and affect regulation (Nakashiba et al., 2012).

## 3 Aging and adult hippocampal neurogenesis

Newly generated neurons progress through dynamic stages important for their normal functioning, finally resulting in behavioral modulation with their integration into hippocampal circuitry. This complex process is regulated by various factors that can increase or decrease neurogenesis, leading to alterations in both the number and function of newly generated neurons (Denoth-Lippuner and Jessberger, 2021). For example, aging is a major physiological factor that contributes to the decline of adult hippocampal neurogenesis by pushing the neural stem cell pool into a quiescent stage (Jiménez Peinado and Urbach, 2023) and reducing the ability of neural stem cells to proliferate (Li and Han, 2025).

Even if the neural stem cells produce new neurons, aging impairs the survival and integration of these newborn neurons into existing circuits (Wu et al., 2023). Indeed, aging disrupts the dentate gyrus microenvironment by reducing synaptic density and compromising vascularization, ultimately creating a less supportive niche for neurogenesis (Cole et al., 2022). The dentate gyrus plays a critical role in pattern separation and episodic memory, and age-related reductions in hippocampal neurogenesis have been directly linked to cognitive decline; studies show that diminished neurogenesis contributes to impairments in spatial learning, memory precision, and cognitive flexibility, all hallmark features of age-related cognitive decline (Kempermann et al., 2018; Moreno-Jiménez et al., 2019). Similarly, adult born neurons are markedly reduced in patients with Alzheimer’s disease, and this reduction correlates with disease progression and cognitive decline (Moreno-Jiménez et al., 2021).

## 4 Stress and adult hippocampal neurogenesis

Another important factor that decreases adult hippocampal neurogenesis is chronic stress, which has been shown to impair the proliferation, survival, differentiation, and maturation of neural progenitor cells in the dentate gyrus (Schoenfeld and Gould, 2012). It reduces the number of newborn neurons through multiple mechanisms, including elevated stress hormones and the actions of inflammatory cytokines and glucocorticoids (Schoenfeld and Gould, 2012). Prolonged exposure to stress leads to elevated levels of stress hormones, particularly cortisol in humans and corticosterone in rodents, which negatively impact neural progenitor proliferation and survival in the dentate gyrus (Saaltink and Vreugdenhil, 2014). Additionally, stress increases neuroinflammation, which in turn diminishes adult neurogenesis by altering the microenvironment of the hippocampal neurogenic niche similarly to alterations observed in aging (Leschik et al., 2021).

Elevated levels of pro-inflammatory cytokines, such as IL-1β, TNF-α, and IL-6, disrupt the proliferation and survival of neural progenitor cells while also impairing their differentiation into functional neurons (Leschik et al., 2021). Even if these newborn cells survive, chronic stress can disrupt maturation and integration of them into the neural circuit (Dioli et al., 2019). These disruptions in adult-born neuron development have been strongly implicated in the emergence of stress-induced behavioral phenotypes, including anhedonia, increased anxiety-like behavior, and cognitive inflexibility, which are all core features of depression-like states in rodents.

## 5 Antidepressants and adult hippocampal neurogenesis

Given the detrimental effects of chronic stress on adult hippocampal neurogenesis and its association with stress-induced behavioral phenotypes, there has been growing interest in the capacity of antidepressant treatments to restore or enhance neurogenesis. Antidepressants have been found to reverse the detrimental effects of chronic stress by enhancing neurogenesis (Dranovsky and Hen, 2006). Indeed, it is shown that disrupting hippocampal neurogenesis blocked the behavioral effects of antidepressants in mice, suggesting that the generation of new neurons in the hippocampus is required for these drugs to exert their therapeutic effects (Santarelli et al., 2003).

Early work by Dranovsky and Hen (2006) synthesized findings across multiple studies, highlighting the role of monoaminergic and serotonergic systems in mediating antidepressant-induced neurogenesis and counteracting stress-related impairments. Consistent with this, Li et al. (2004) reported that the classical antidepressant moclobemide (MOC) reversed NMDA-induced suppression of neurogenesis and increased progenitor cell proliferation in the dentate gyrus, even under chronic stress conditions. Similarly, Czéh et al. (2001) demonstrated that chronic administration of the tricyclic antidepressant tianeptine restored neurogenesis and hippocampal volume following exposure to chronic unpredictable stress. Supporting a mechanistic role for serotonergic signaling, Diaz et al. (2012) found that the pro-neurogenic effects of selective serotonin reuptake inhibitors (SSRIs) such as fluoxetine and paroxetine required intact 5-HT2B receptor function: neurogenesis was abolished in 5-HT2B receptor knockout mice or following pharmacological inactivation of the receptor. More recent work has extended these findings to atypical antidepressants. Rawat et al. (2024) showed that both acute and chronic ketamine treatment enhanced neurogenesis, but via distinct mechanisms: a single dose increased newborn neuron activity, whereas repeated dosing promoted neural proliferation.

## 6 Other factors that may affect hippocampal neurogenesis

Beyond pharmacological interventions, increasing the number of newborn neurons through environmental enrichment or physical exercise has been shown to promote stress resilience and improve behavioral outcomes (Fölsz et al., 2023). This is in direct contrast to common drugs of abuse such as alcohol, as multiple studies have shown disruption of cell proliferation and survival in rodent models of alcohol abuse, highlighting a potential mechanism for the neurodegeneration and cognitive impairments commonly seen in alcohol abuse disorders (Nixon and Crews, 2002; He et al., 2005; Morris et al., 2010; Taffe et al., 2010). While the precise pathways through which alcohol exposure disrupts neurogenesis are unknown, strong evidence suggests astrogliosis, metabolic disruptions, and proteasomal impairments are involved (Kelso et al., 2011; Liu et al., 2022). Analyses of postmortem human tissue corroborated prior animal studies and revealed a marked depletion of neural stem/progenitor cells alongside reductions in cell proliferation and maturation within the dentate gyrus (Le Maître et al., 2018). Opioids similarly disrupt multiple aspects of neurogenesis while also shifting dentate gyrus neural phenotypes toward a more inhibitory, GABAergic identity (Zhang et al., 2016a,b; Kahn et al., 2005). While opiates are generally found to reduce cell proliferation in the hippocampus at multiple stages of development, their effects on cell survival are less understood and potentially even beneficial (Wu et al., 2014; Pettit et al., 2012). Zhang et al. (2016a) present a thorough review of opioids’ effects on neurogenesis by highlighting changes to neural progenitors and potential consequences for memory formation and modulation of rewards pathways.

The relationship between neuroinflammation and neurogenesis is complex and varies greatly depending on the exact nature of the immune response. Microglia, astrocytes, and the signaling molecules they secrete can have both anti- and pro-neurogenic effects on virtually every step in the process; from initial proliferation to migration and maturation, and finally cell survival (Amanollahi et al., 2023). While there is not a single, unified neurogenic response to inflammation in either direction, it is clear that these processes are intimately connected. Future studies should place a strong emphasis on better understanding the specific immune states that drive concerted increases or decreases to neurogenesis.

## 7 Neural activity of newborn neurons

While increasing the number of newborn neurons in the hippocampus is crucial for maintaining cognitive function and resilience to stress, the activity and functional integration of these neurons are equally important. Newly generated neurons must establish appropriate synaptic connections and receive proper excitatory and inhibitory inputs to contribute to hippocampal function. However, while much research has focused on factors that influence the production and survival of these neurons, relatively little is known about how their activity is regulated and how disruptions in their functional maturation may contribute to hippocampal dysfunction.

Ge et al. (2007) studied the electrophysiological characteristics of newly generated adult neurons in the dentate gyrus using retrovirus-mediated birthdating and labeling. They observed a distinct period of cell age between 1 and 1.5 months in which new neurons demonstrated both increased long-term potentiation (LTP) amplitude and decreased LTP induction threshold, positing that this allows adult newborn neurons to play a role beyond the straightforward replacement of existing mature neurons (Ge et al., 2007). The high intrinsic excitability of immature dentate gyrus cells allows them to effectively transduce signals from weaker inputs; this represents a potential compensatory mechanism intended to balance the firing activity between mature and immature hippocampal neurons (Jessberger and Kempermann, 2003; Mongiat et al., 2009). Rather than directly modulating mature dentate granule cells, adult-born neurons are integrated upstream to gate the flow of information to the hippocampus proper; the functional impact of neurogenesis in the hippocampus is mediated through local inhibitory interneurons that inhibit the mature granule cells in the dentate gyrus (Drew et al., 2016). By stimulating adult-born neurons in awake, behaving animals, the researchers demonstrated that increasing the activity of these neurons reduced the overall activation of mature granule cells during exploration of a novel environment (Drew et al., 2016). Additionally, stimulating adult-born neurons in the dentate gyrus refines hippocampal activity by increasing population sparsity, whereas their suppression increases principal cell firing rates and disrupts novel object recognition (McHugh et al., 2022). This suggests that adult-born neurons play a role in controlling the balance of excitation and inhibition in the dentate gyrus, which could be crucial for processes like memory formation and cognitive flexibility (Drew et al., 2016). However, the relationship between neural activity and neurogenesis is complex and bi-directional; local network activity drives maturation of adult-born neurons, which are then capable of modulating local network activity themselves (Piatti et al., 2011; Li et al., 2023a). For example, the maturation of newborn neurons is modulated by local network activity along the septotemporal axis of the hippocampus, where higher basal neuronal activity of the septal dentate gyrus leads to faster newborn neuronal development compared to the temporal dentate gyrus (Piatti et al., 2011). On the other hand, voluntary exercise selectively enhances temporal dentate gyrus network activity, accelerates maturation of newborn neurons, and suppresses these septotemporal differences. Interestingly, reducing the intrinsic excitability of these newborn neurons delays their maturation, even in an active network (Piatti et al., 2011). Therefore, both extrinsic network activity and cell-intrinsic excitability modulate the pace of newborn neuronal development in the dentate gyrus, which in turn affects their contribution to the dentate gyrus circuitry.

Activity-based regulation of adult hippocampal neurogenesis is not limited to local hippocampal circuits; the hypothalamic supramammillary nucleus (SuM) promotes activation and proliferation of radial neural stem cells through glutamatergic inputs to the dentate gyrus, while GABAergic inputs from the SuM leads to maturation of newborn neurons (Li et al., 2022). Moreover, chronic patterned optogenetic stimulation of SuM neurons increases the number and maturity of newborn dentate gyrus neurons, resulting in enhanced memory and reduced anxiety-like behavior, while SuM ablation impairs both neurogenesis and behavioral outcomes (Li et al., 2022). Interestingly, in a mouse model of Alzheimer’s disease, this same SuM-enhanced generation and function of adult-born neurons was also shown to ameliorate deficits in both memory and affect (Li et al., 2023b). These findings show that long-range, activity-dependent inputs from the SuM to the dentate gyrus can exert circuit-specific control over adult-born neuron activity, maturation, and function, thereby shaping hippocampal contributions to cognition and emotion across both healthy and diseased states. Therefore, it is not only the number and activity of these adult-born neurons that influence hippocampal circuitry and behavior, but also the specificity and strength of their synaptic connections.

Building on this framework, other studies have shown that the behavioral effects of antidepressant treatments also depend on the activity of newborn neurons rather than solely on their number. For example, the rapid antidepressant effects of a single ketamine dose are due to increased activity of immature neurons in the hippocampal dentate gyrus without requiring an increase in the number of newborn neurons (Rawat et al., 2024). It is also shown that inhibition of newborn neuronal activity abolished the antidepressant effects of fluoxetine (Tunc-Ozcan et al., 2019). Conversely, direct activation of newborn neurons without altering neurogenesis is sufficient to produce antidepressant-like effects, suggesting that it is not merely the increase in neuron numbers but their activity that is essential for mood regulation (Tunc-Ozcan et al., 2019). Moreover, silencing of newborn neurons in the ventral dentate gyrus increased the activity of mature granule cells and made the mice more susceptible to stress, while increasing neurogenesis or inhibiting dentate gyrus activity promoted resilience to stress (Anacker et al., 2018).

Accumulating evidence suggests that adult-born hippocampal neurons are involved in information processing, but the role of neuronal activity in how and when these immature neurons are incorporated into local circuitry is less clear. We do understand, however, that the functional contribution of adult born neurons to hippocampal circuitry depends not only on their generation and survival, but also on their precise integration, excitability, and responsiveness within existing neural networks. Future studies that investigate how adult-born neurons are integrated into local circuits are necessary to better understand the reciprocal, activity-dependent nature of hippocampal neurogenesis.

## 8 Discussion

**FIGURE 2:**
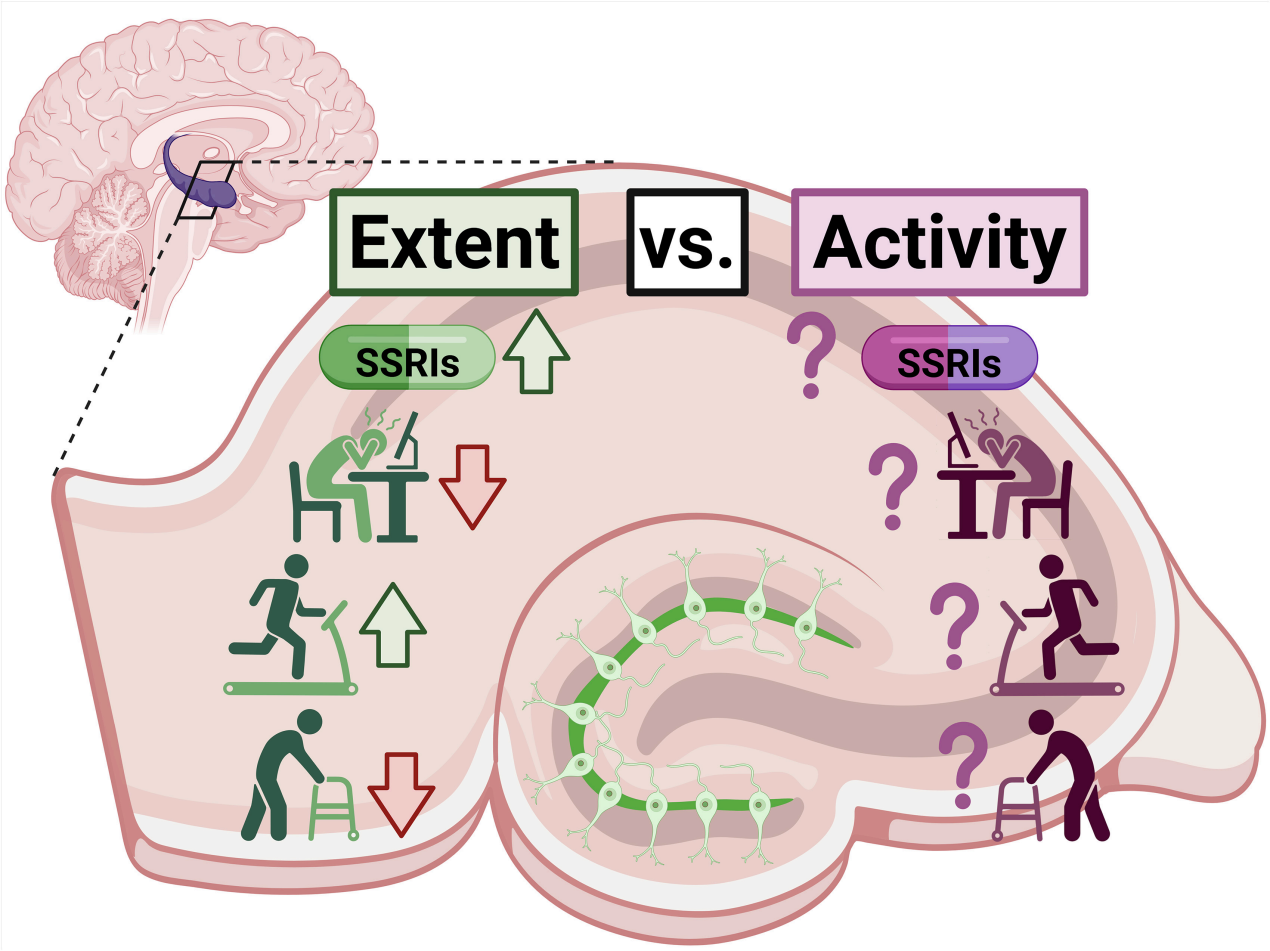
Current knowledge and outstanding questions for extent versus activity of adult hippocampal neurogenesis. Extensive evidence on the extent of neurogenesis shows increases because of SSRIs and exercise but decreases due to chronic stress and aging. Comparatively limited research has focused on how the activity of these newborn neurons is affected by the same variables. Created in BioRender. Betters, R. (2025) https://BioRender.com/qs3hm52.

Adult hippocampal neurogenesis is a dynamic and highly regulated process essential for cognitive flexibility, affective regulation, and resilience to stress. Its decline with aging and disruption by chronic stress is closely linked to impairments in memory, mood, and behavioral adaptability. Conversely, SSRIs and physical exercise increase neurogenesis and ameliorate these impairments, potentially through shared mechanisms (Figure 2). While numerous studies have focused on increasing the number of newborn neurons through pharmacological and environmental means, accumulating (albeit limited) evidence highlights that their functional maturation and activity-dependent integration into hippocampal circuits are equally critical. The capacity of these neurons to modulate local network excitability and contribute to behavioral outcomes underscores their unique role in hippocampal processing. Understanding how both neurogenesis and newborn neuron activity are regulated across lifespans, in disease, and in response to therapeutic interventions, will be key to developing more effective strategies for treating neurological disorders. More research is needed on how the activity of newborn neurons is affected by aging and environmental factors to better understand the full picture of human neurogenesis (Figure 2).
